# Thick-Film Carbon Dioxide Sensor via Anodic Adsorbate Stripping Technique and Its Structural Dependence

**DOI:** 10.3390/s90907203

**Published:** 2009-09-09

**Authors:** Kanokorn Photinon, Shih-Han Wang, Chung-Chiun Liu

**Affiliations:** 1 Chemical Engineering Department, Case Western Reserve University, 10900 Euclid Ave., Cleveland OH 44106, USA; E-Mail: cxl9@case.edu (C.C.L.); 2 Chemical Engineering Department, I-Shuo University, 1, Sec. 1, Syuecheng Rd., Dashu Township, Kaohsiung County 840, Taiwan; E-Mail: shwang@mail.isu.edu.tw (S.H.W.)

**Keywords:** carbon dioxide sensor, anodic adsorbate stripping, thick-film, platinum nanoparticles, structural dependence

## Abstract

A three-electrode based CO_2_ sensor was fabricated using thick-film technology. The performance of this sensor was further enhanced by incorporating platinum nanoparticles onto the working electrode surface. An eight-fold increase in the signal output was obtained from the electrode with the platinum nanoparticles. The sensing output was linearly related to the CO_2_ presented. Stability measurements demonstrated that the decline of the active surface area and the sensitivity of the sensor were 8% and 13%, respectively, over a two week period of time. The sensor response appeared to be a structural dependence of the crystallographic orientation of platinum electrode.

## 1. Introduction

Monitoring and control of carbon dioxide (CO_2_) in the atmosphere are needed due to heightened environmental concerns. Consequently, the development of reliable CO_2_ monitoring techniques is scientifically and economically important. Various CO_2_ analytical methods are currently utilized in laboratory experiments and field observations. Infrared sensors, conductivity sensors, and Severinghaus-type sensors (sensors based on a glass pH electrode covered with a glass permeable membrane) for the detection of CO_2_ are commercially available [1]. However, these sensors have limitations due to the interferences and the non-linear response to CO_2_ content. With well-defined oxidation and reduction potential for the particular species, amperometric sensors can be used to minimize interferences. Amperometric CO_2_ sensors have been developed for CO_2_ sensing and studied extensively, but a complication with hydrogen evolution has prevented amperometric CO_2_ sensors from reaching their commercial potential. The approach of altering the electrochemistry of CO_2_ by reacting it with selected complex species [2] has also been unsuccessful in practical CO_2_ sensing. An anodic adsorbate stripping technique of the adsorbed CO_2_ was developed to provide a linear dependence on CO_2_ content. The technique was based on forming adsorbed CO_2_ and then oxidizing the previously formed adsorbate. The CO_2_ product that adsorbs at low potential will be referred to as Pt-HCOO throughout this manuscript. Although the true structure of the Pt-HCOO is unclear, its linear response is sufficient for quantitative study as a Pt–HCOO sensor in our specific applications.

The adsorption of carbon dioxide on platinum (Pt) electrodes from acid solutions has been known for more than 40 years. In acidic medium, CO_2_ only adsorbs onto hydrogen previously adsorbed on a platinum surface (Pt–H). Numerous investigations and studies had been carried out on this phenomenon, but the structure of Pt–HCOO was not consistent and had been the subject of controversy [3–12]. According to the results obtained with various nonelectrochemical techniques (IR, mass spectrometry, radiochemical methods), the following structures of the Pt–HCOO radicals were proposed: –COOH [5–7,13,14], –COH [8,9,14], –HCO [12], linearly or bridged bonded –CO [11,15–18]. The investigation of the mechanism and the Pt–HCOO radicals are beyond the scope of this work. Nevertheless, it is feasible to detect the Pt–HCOO on a platinum surface in an acidic medium as a means to quantify CO_2_ in a gaseous test environment, regardless of any knowledge about the true structures of the Pt–HCOO radicals. The relationship between the oxidation current and the CO_2_ content is linear, suggesting a very desirable and sensitive CO_2_ detection.

Various manufacturing processes can be used to fabricate an electrochemical based CO_2_ sensor. In this study, the CO_2_ sensor prototype was fabricated using a thick-film screen printing technique. Thick-film screen printing produces geometrically well-defined, highly reproducible sensor structures in a cost-effective manner. The sensor operated in the anodic adsorbate stripping mode, and the platinum nanoparticles were incorporated onto the working electrode enhancing the sensor performance. Our sensor can thus offer a device for CO_2_ detection with high performance and at a relatively modest cost.

## 2. Results and Discussion

### 2.1. Anodic Adsorbate Stripping Response

**Figure 1.**
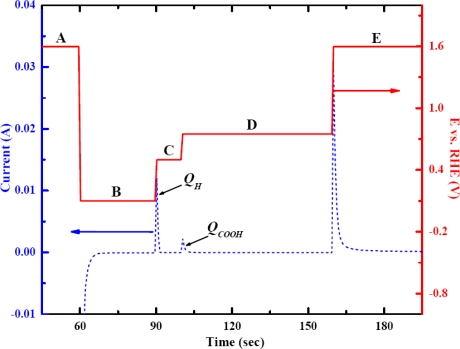
Potential step program and i–t curve response for the anodic stripping technique, ( 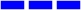) i–t curve response, ( 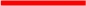) potential step program.

The potential stepped program is illustrated in Figure 1. The adsorption of CO_2_ on Pt started when the potential was stepped down (step B) to the adsorbed hydrogen potential. Once the potential was changed to where oxidation of the remaining Pt–H occurs (step C), the anodic current increased accordingly. The contribution of the anodic current from the oxidation of Pt–HCOO took place when its oxidation potential was reached; in this case, the oxidation potential was +0.75 V (step D). The oxidation charged for Pt–HCOO (Q_HCOO_) could then be determined from the area under the curve from this step. Finally, large anodic current was produced from the formation of surface oxides when the potential was stepped to +1.60 V (step E).

**Figure 2.**
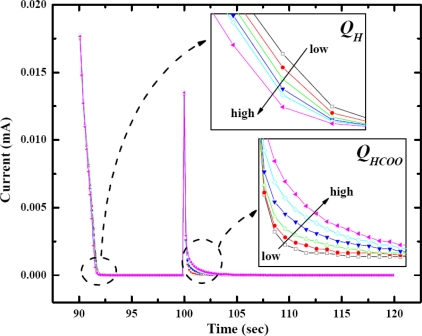
Anodic stripping response for various %CO_2_ in gas sample on a Pt–nano electrode demonstrating the inverse relationship between *Q_H_* and *Q_HCOO_*, ( 
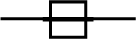
) 0.0% CO_2_, ( 
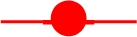
) 0.5% CO_2_, ( 
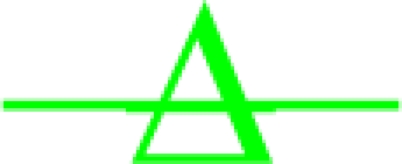
) 1.0% CO_2_, ( 
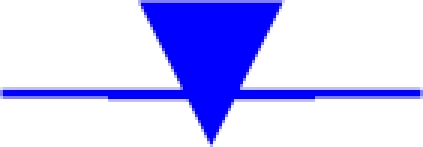
) 2.0% CO_2_, ( 
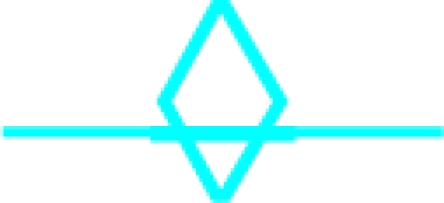
) 3.0% CO_2_, ( 
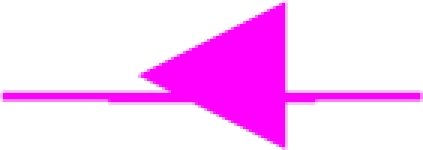
) 5.0% CO_2_.

**Figure 3.**
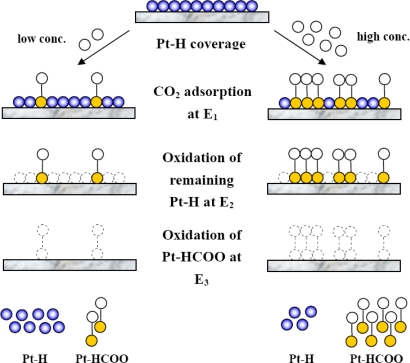
Schematic of anodic stripping of CO_2_ at low and high concentration illustrating the inverse relationship between *Q_H_* and *Q_HCOO_*.

Also depicted in Figure 1 is an i–t curve which illustrates the oxidation charge of the remaining Pt–H (*Q_H_*) and the oxidation charge of Pt–HCOO (*Q_HCOO_*), defined from each area under the corresponding peak. The first peak starting at 90 s represented the oxidation of the remaining Pt–H, while the second peak starting at 100 s was from the Pt–HCOO oxidation. CO_2_ adsorption occurred only in the presence of Pt–H, hence the amount of Pt–H consumed is correlated to the amount of CO_2_ that could be adsorbed. A higher amount of Pt–HCOO resulted in higher consumption of Pt–H, therefore the remaining Pt–H decreased accordingly as shown in Figure 2. The insets of Figure 2 demonstrate the inversely proportional relationship between two oxidation charges. The schematic of anodic adsorbate stripping of CO_2_ at low and high concentration illustrating the inverse relationship between *Q_H_* and *Q_HCOO_* is shown in Figure 3. Typically, only *Q_HCOO_* was of interest for the study of CO_2_ adsorption using the anodic stripping [19,20]. However, the inversely proportional relationship between *Q_H_* and *Q_HCOO_* was found to be essential and informative to explain the dependence of the sensitivity on adsorption potential.

### 2.2. Signal Enhancement using Pt Nanoparticles

**Figure 4.**
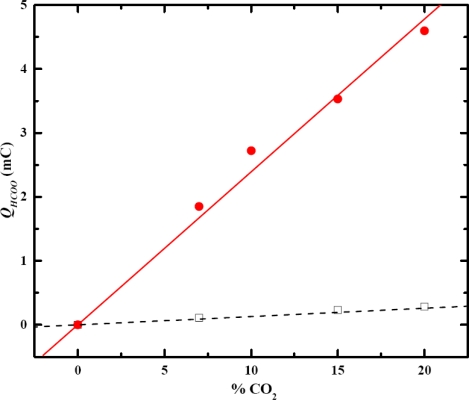
Calibration curves for CO_2_ measurement from chronoamperometry at +0.75 V versus RHE, sensitivity of Pt–nano electrode is eight times higher than that of Pt–Au electrode, ( 
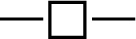
) Pt–Au, ( 
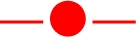
) Pt–nano.

The calibration curves for CO_2_ measurement are shown in Figure 4; the sensing property was found to be enhanced eight-fold employing Pt nanoparticles. The sensitivity for the Pt–Au electrode was 0.030 mC/(%CO_2_), and for the Pt–nano electrode, 0.239 mC/(%CO_2_). The linearity of the calibration curves was excellent with R^2^ > 0.993. The substantial current increase was due to the increase surface area of the Pt nanoparticles. The ratio of active surface area to geometrical area for Pt–Au and Pt–nano electrodes were 1.56 and 20.80, respectively. Consequently, the sensitivity improvement for CO_2_ detection was anticipated.

### 2.3. Calibration of the Sensor Prototype and Interference Study

**Figure 5.**
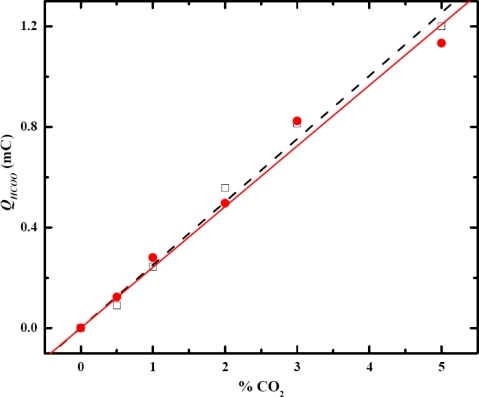
Calibration curves Response of CO_2_ sensor when the sample is balance with, ( 
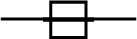
) air, ( 
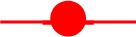
) argon; there is no significant effect from other gases present in ambient air on CO_2_ measurement.

A potential application for the CO_2_ sensor developed and assessed in this research would be environmental monitoring. Atmospheric air contains 0.038% CO_2_, while the concentration of CO_2_ in exhaled air is approximately 6%. The OSHA (Occupational Safety and Health Administration) regulations limit CO_2_ in the workplace to 0.5% for a prolonged period, and the U.S. National Institute for Occupational Safety and Health limits brief exposures (up to ten minutes) of CO_2_ to 3%. The detection limit of our sensor prototype is 0.25% CO_2_. Thus, the sensing performance of this CO_2_ sensor was investigated in the lower range (less than 5%). Five compositions of CO_2_, 0.5%, 1.0%, 2.0%, 3.0% and 5.0%, were prepared using a gas mixture and tested and the corresponding sensor responses are shown in Figure 5. The sensitivity of the sensor for detecting CO_2_ was 0.249 mC/(%CO_2_). The calibration curves had an excellent linearity with R^2^ > 0.994.

Since the gas sample for the sensor was atmospheric air containing oxygen, nitrogen and other gases in addition to CO_2_, it is thus necessary to investigate for possible interferences from the other gases. Argon was used to balance the CO_2_ mixture in the test gas medium instead of air. The sensitivity was found to be 0.240 mC/(%CO_2_) which was only 3.61% different from the air-balanced gas samples. A good linearity of the calibration curves with R^2^ > 0.992 was obtained. Therefore, the sensor response of this developed CO_2_ sensor demonstrated no significant interference from other gases in ambient air. This is depicted in Figure 5 as well.

The other possible interferences are carbon monoxide (CO) and organic compounds in the case of harsh environments, which was considered being outside the scope of this work. Nevertheless, since CO and organics can adsorb on a Pt surface without adsorbed-H and the oxidation of those molecules is irreversible [21], therefore in case of CO and organics presenting in the system, those molecules could be readily oxidized at potentials above H-adsorption and below surface oxide formation to get rid of any interferences prior to the measurement of CO_2_.

### 2.4. Sensor Stability

The stability of a CO_2_ sensor is considered one of its key performance characteristics. In the case of thick-film printing production, good short term stability would be expected rather than in the long term. The active surface area of the sensing electrode elements can be used as an indication of the stability of the sensor. Thus, the measurement of active area of the working electrode was taken after operating the sensor for eight hours. The measurement was carried out on a periodic basis for 14 days. We found that the active electrode surface area continuously declined over the testing period. After 14 days, the active area of the working electrode was approximately 8% less than that of the beginning of the evaluation (result not shown). The stability of this sensor was considered acceptable for thick-film gas sensor, whose other aspects could readily mitigate its moderate stability.

The dependence of the sensitivity on the stability of this CO_2_ sensor was also studied. A strong dependence was intuitively expected from the fact that sensor response would be proportional to the electrode surface area. The sensor responses for different CO_2_ content in gas sample on Day 1 and Day 14 were compared. The sensitivities of the sensor for Day 1 and Day 14 were 0.235 and 0.205 mC/(%CO_2_), respectively. The reduction of sensitivity was less than 13% (result not shown).

### 2.5. Structural Dependence on Sensing Property

**Figure 6.**
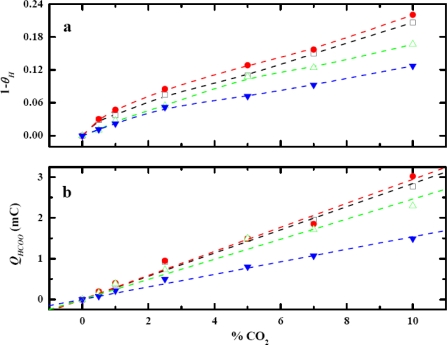
Dependence of (a) *1-θ_H_* and (b) *Q_HCOO_* of Pt–nano electrode on adsorption potential, highest *Q_HCOO_* and *1-θ_H_* is at 0.10 V versus RHE ( 
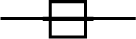
) 0.15 V, ( 
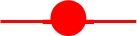
) 0.10 V, ( 
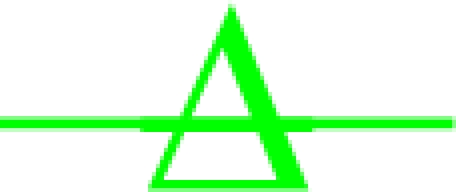
) 0.05, ( 
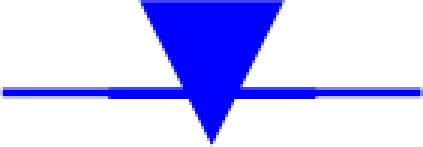
) 0.00 V.

Since the potential for CO_2_ to adsorb is in the voltage window of hydrogen adsorption potential (0.00 V to 0.40 V versus RHE), an investigation was carried out to obtain the optimum adsorbed potential and the dependence of the quantity of Pt–H on the adsorbed potential. The amount of the Pt–H produced strongly depends on the adsorption potential as anticipated (result not shown). At a more negative adsorption potential, a higher yield of Pt–H was obtained. Experimentally, the range of the adsorption potential between 0.00 V to 0.15 V versus RHE was employed. Figure 6(a) shows the behavior of fraction of Pt-H being consumed, (1 – *θ_H_*), at different CO_2_ concentrations.

(1) $$\theta_{H}=Q_{H}/Q_{H}^{0}$$

(2) $$1-\theta_{H}=\left(Q_{H}^{0}-Q_{H}\right)/Q_{H}^{0}$$

where $Q_{H}^{0}$ is the oxidation charge of Pt–H produced when there is no CO_2_ present at the corresponding potential. *Q_H_* is the oxidation charge of remaining Pt–H that are not consumed in CO_2_ adsorption reaction. Figure 6(b) shows the calibration curve for each adsorption potential.

**Figure 7.**
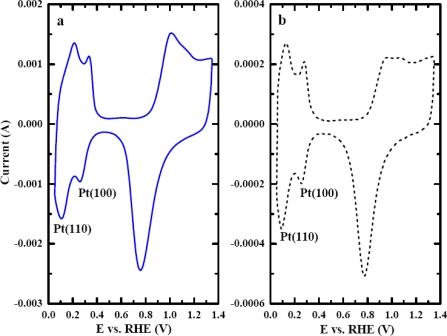
Voltammograms of (a) Pt–nano and (b) Pt–Au electrodes in 0.10 M H_2_SO_4_ using a scan rate of 50 mV/s, voltammogram resembles the superimposition of three main crystallographic planes. Only peaks from the most two dominant planes revealed; Pt(110) and Pt(100).

The dependence of the sensitivity of this CO_2_ sensor illustrated in Figure 6(b) was not exactly as anticipated. The order of the sensitivity from high to low was 0.10 V > 0.15 V > 0.05 V > 0.00 V versus RHE. Figure 6(a) showed that the ratio of consumed Pt–H was the highest at 0.10 V, despite having less Pt–H produced than at 0.05 V and 0.00 V. The results from Figure 6(a) suggest that the portion of the Pt–H consumed during the CO_2_ adsorption plays a key role for the sensitivity dependence on the adsorption potential. This particular portion of the Pt–H consumed was more reactive than the rest of Pt–H on the electrode surface. It was more favorable for CO_2_ to adsorb on these reactive sites than on the other sites. Evidently, the reactive Pt–H was produced most at 0.10 V because the consumption was the highest. *Q_H_* proved to be the pivotal information needed to obtain the observation about the reactive Pt–H preferentially produced at 0.10 V. *Q_HCOO_* alone was clearly not sufficient to explain the unconventional dependence of the sensitivity on the adsorption potential. The sensor response, hence the sensitivity, at this potential was anticipated to be the highest. A rationale for this observation was made from the dependence of CO_2_ adsorption on the crystallography of platinum. The polycrystalline Pt nanoparticles consist of three main crystal planes that are of the interest in this study: Pt(110), Pt(100), and Pt(111). The Pt(111) and Pt(100) surface has flat structures, while Pt(110) has step sites that are found to be more reactive to CO_2_ adsorption than the other two planes [10,22–26]. The structural model also shows that the step sites of Pt(110) creates a highly coordinated configuration that would be more favorable for atoms or molecules to reside, making Pt(110) more reactive than Pt(111) and Pt(100) [27]. Polycrystalline platinum had been studied using CV [25] and the results showed that the voltammograms exhibited a superimposition of all crystal planes. The CV voltammogram of a Pt–nano electrode in 0.1 M H_2_SO_4_ is shown in Figure 7(a) and that of a Pt–Au electrode in the same medium is shown in Figure 7(b). These results exhibited a similar pattern and it was consistent with other reported results [25]. The peak at 0.10 V resembles the Pt(110) pattern which produces the most reactive Pt–H. Therefore, when the electrode was polarized at 0.10 V, the more reactive Pt–H was produced resulting in higher sensor response. This experimental result served well as the rationale for the dependence of sensitivity of the CO_2_ sensor.

**Figure 8.**
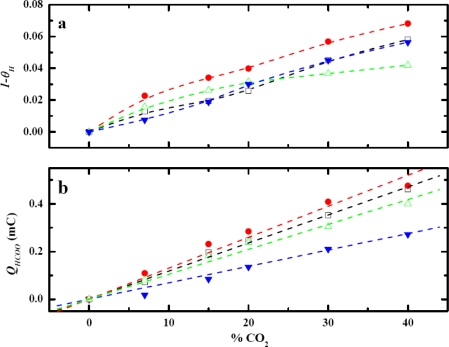
Dependence of (a) *1-θ_H_* and (b) *Q_HCOO_* of Pt–Au electrode on adsorption potential, highest *Q_HCOO_* and *1-θ_H_* is at 0.10 V versus RHE ( 
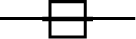
) 0.15 V, ( 
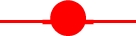
) 0.10 V, ( 
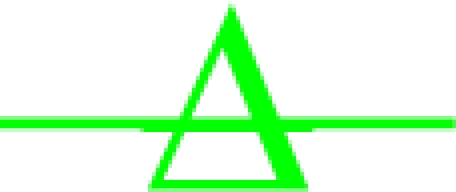
) 0.05, ( 
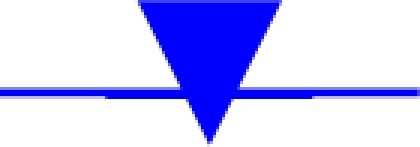
) 0.00 V.

In order to further support this conclusion, studies on the influence of adsorbed potential on CO_2_ adsorption using a Pt–Au electrode were performed. Although the current output of Pt–Au was approximately one fifth of the Pt–nano electrode, the Pt–Au electrode also exhibited a similar pattern voltammogram in a 0.10 M H_2_SO_4_ in Figure 7(b). Thus, the behavior of the sensor response and the sensitivity of the Pt–Au electrode were expected to be analogous to that of the Pt–nano electrode. As was anticipated, a similar effect of adsorption potential on calibration curves was found and is shown in Figure 8. The sensitivity of the sensor was the highest at the adsorption potential of 0.10 V. The sensitivity of this Pt–Au electrode from high to low was in the same order of the Pt–nano electrode, i.e., 0.10 V > 0.15 V > 0.05 V > 0.00 V. The results from the Pt–Au were consistent with the results obtained from the Pt–nano electrode. Also, reactive Pt–H was produced and consumed the most at 0.10 V, which explained the sensitivity dependence on the adsorption potential of the Pt–Au electrode.

## 3. Experimental Section

### 3.1. Sensor Fabrication

**Figure 9.**
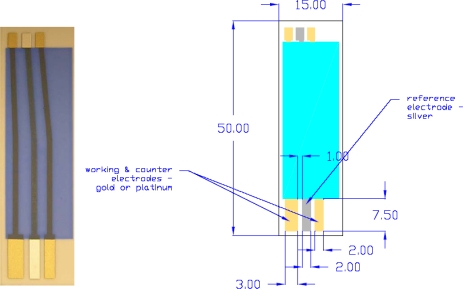
Three-electrode (Au/Ag/Au) sensor layout and its dimensions (in mm).

**Figure 10.**
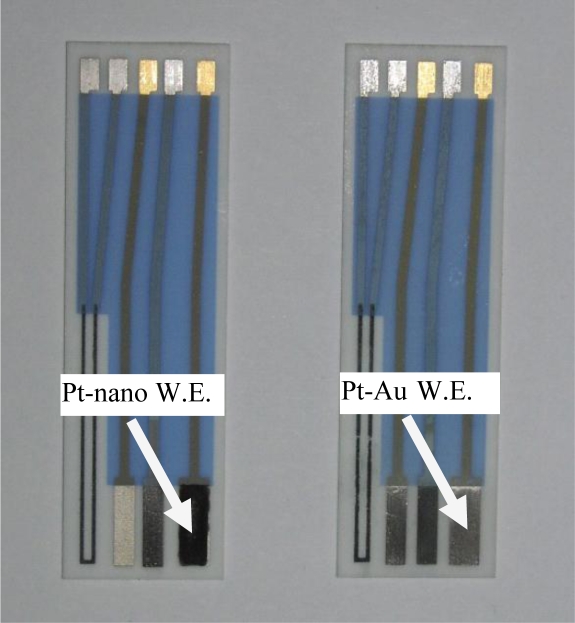
Three-electrode sensor prototypes after platinization, chloridization and hand-printing of Pt nanoparticles ink.

The sensor (Figure 9) was comprised of three electrodes: gold/silver/gold (Au/Ag/Au), and was fabricated by thick-film screen printing. The substrate for the sensor prototype was 96% alumina (Coors, Golden, CO, USA). The metal inks that were used to print the metal leads or electrodes are commercially available. Gold layers were printed first, followed by the silver and the insulation layers. After each layer was printed, the solvent was burnt off at 110 °C and the substrate was fired at 850 °C under an ambient air atmosphere. The gold electrode served as the base for the construction of the platinized gold (Pt–Au) or platinum nanoparticles (Pt–nano) working electrodes and the platinized gold counter electrode. Pt–Au electrode was formed by electroplating the thick-film gold electrode in a 5% hexachloroplatinic acid (H_2_PtCl_6_, Sigma-Aldrich, St Louis, MO, USA). To platinize gold electrode, an applied potential of −1.0 V (versus a Pt gauze) was used and the plating process was approximately 180 seconds. To prepare Pt nanoparticle ink, 3% w/v hydroxyethyl cellulose (HEC) (Sigma-Aldrich) was first dissolved in 2 mL DI water, and then 6% w/v polyethylenimine (PEI) (Sigma-Aldrich) was added. Pt nanoparticles in the average diameter of 5.5 nm (E-TEK) of 10% w/v was added and mixed well using a homogenizer. This Pt nanoparticle ink was the hand-printed on the Pt–Au working electrode surface forming a 2 mm × 7.5 mm geometric surface area. The sensor was dried for 1 hour and then heated at 125 °C for 3 hours in an oven under an ambient air atmosphere. The formation of the AgCl layer on the Ag base electrode was achieved electrochemically in a 0.10 M HCl aqueous solution. A potential of 0.50 V (versus a Pt wire gauze) was applied for 90 seconds. This resulted in a three-electrode based sensor with either a Pt–Au or a Pt–nano working electrode, a Pt–Au counter electrode and a Ag/AgCl reference electrode (Figure 10). All potentials in this paper are referred to RHE.

### 3.2. Anodic Adsorbate Stripping Technique

**Figure 11.**
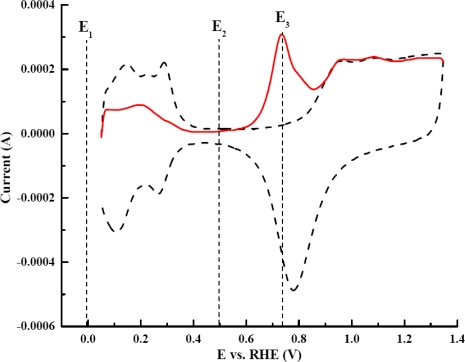
Cyclic voltammograms of ( 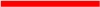) 100% CO_2_, and (---) 0% CO_2_ in 0.10 M H_2_SO_4_ using a scan rate of 50 mV/s, showing the potential range where Pt–HCOO is formed and oxidized.

As Pt–HCOO has to be formed at a low potential where Pt–H is available before undergoing oxidation at higher potential, potential has to be programmed accordingly. From a cyclic voltammogram (Figure 11), the Pt–HCOO is formed in the voltage window of 0.00 to 0.50 V versus RHE (voltage range of E_1_ and E_2_). The oxidation of the Pt–HCOO takes place at 0.75 V (E_3_). Thus for a CO_2_ sensor, the platinum working electrode is first held at 0.00 V for approximately 60 seconds. When a potential of 0.75 V was applied, the oxidation of Pt–HCOO took place and the current produced could then be used to quantify the CO_2_ presented. The electrolyte for all experiments was 0.10 M H_2_SO_4_.

### 3.3. Sensing Performance and Interference Study

To evaluate the sensor performance, the relationship between the sensor outputs and the CO_2_ concentrations needed to be established. The sensitivity and the reproducibility of this sensor prototype in the CO_2_ range of 0%–20% were determined. The effect of possible interference by other gases presented in ambient air was also assessed. The main components of ambient air are N_2_ and O_2_. Therefore, the results from the samples balanced with air and argon were compared in order to investigate the effect of the interferences.

### 3.4. Sensor Stability

The sensor stability was investigated on a periodic basis for 14 days by comparing the active surface area of the working electrode, which could be used as an indication for the stability of the sensor since the sensor output strongly depends on the surface area of the electrode. The active surface area was calculated from the area under the voltammogram in the hydrogen adsorption region with the appropriate corrections [4,19]. After the sensor was operated for at least eight hours, the voltammogram was recorded and the active surface area was determined. The dependence of the sensor performance on its stability was also studied. The sensitivity of the sensor on Day 1 and Day 14 were compared and the effect of the stability on the sensor was then assessed.

## 4. Conclusions

Our thick-film sensor developed for CO_2_ detection showed promising performance and stability. The sensor response was enhanced by incorporating Pt nanoparticles onto the working electrode surface. The sensitivity for the Pt–nano electrode was eight times higher than that of the Pt–Au electrode. The sensor also demonstrated good sensitivity and reproducibility. A sensitivity of 0.249 mC/(%CO_2_) was obtained. Since nitrogen, oxygen and other gases are also present in the gas sample for the potential application, the effects of these gases on the sensor properties were evaluated. The results showed that the response of the sensor was not interfered by these gases. Over 14 days, the active surface area of the working electrode of the sensor decreased approximately 8% and the sensitivity dropped 13%. Our stability study showed that the sensor would maintain its sensing performance for a reasonably short term use, and the sensor can also be reusable. The thick-film manufacturing technique already offered a cost-effective approach to produce these CO_2_ sensors with acceptable stability and excellent performance.

For structural dependence, it was unexpected that 0.10 V versus RHE yielded the highest sensitivity instead of 0.00 V, where Pt–H produced the most. The crystallographic studies suggested that the quantity of reactive Pt–H formed on Pt(110) was the highest at 0.10 V. This reactive Pt–H of Pt(110) favored CO_2_ adsorption resulting in the highest sensitivity. A similar assessment was obtained for both Pt–nano and Pt–Au electrodes. Despite of having an order of magnitude difference in the current outputs, Pt–nano and Pt–Au yielded analogous patterns of CV voltammograms. Therefore, the dependence of the sensitivity of Pt–nano electrode on the adsorption potential was expected to be consistent with that of the Pt–Au electrode. The presumed statement was confirmed and illustrated by the results of this study.
